# Predictive score for mortality in patients with COPD exacerbations attending hospital emergency departments

**DOI:** 10.1186/1741-7015-12-66

**Published:** 2014-04-23

**Authors:** José M Quintana, Cristóbal Esteban, Anette Unzurrunzaga, Susana Garcia-Gutierrez, Nerea Gonzalez, Irantzu Barrio, Inmaculada Arostegui, Iratxe Lafuente, Marisa Bare, Nerea Fernandez-de-Larrea, Silvia Vidal

**Affiliations:** 1 Unidad de Investigación, Hospital Galdakao-Usansolo, Barrio Labeaga s/n, 48960 Galdakao Vizcaya, Spain; 2 Servicio de Neumologia, Hospital Galdakao-Usansolo, Galdakao, Spain; 3 Departamento de Matemática Aplicada, Estadística e Investigación Operativa, Universidad del País Vasco, Lejona, Bizkaia, Spain; 4 Unidad de Epidemiología Clínica, Corporacio Parc Tauli, Barcelona, Spain; 5 Agencia Lain Entralgo, Madrid, Spain; 6 Unidad de Calidad, Hospital Valme, Sevilla, Spain; 7 Red de Investigación en Servicios Sanitarios y Enfermedades Crónicas (REDISSEC), Bilbao, Bizkaia, Spain

**Keywords:** COPD exacerbations, Mortality, Prediction Rule, Prospective Cohort Study, Risk analysis

## Abstract

**Background:**

Limited information is available about predictors of short-term outcomes in patients with exacerbation of chronic obstructive pulmonary disease (eCOPD) attending an emergency department (ED). Such information could help stratify these patients and guide medical decision-making. The aim of this study was to develop a clinical prediction rule for short-term mortality during hospital admission or within a week after the index ED visit.

**Methods:**

This was a prospective cohort study of patients with eCOPD attending the EDs of 16 participating hospitals. Recruitment started in June 2008 and ended in September 2010. Information on possible predictor variables was recorded during the time the patient was evaluated in the ED, at the time a decision was made to admit the patient to the hospital or discharge home, and during follow-up. Main short-term outcomes were death during hospital admission or within 1 week of discharge to home from the ED, as well as at death within 1 month of the index ED visit. Multivariate logistic regression models were developed in a derivation sample and validated in a validation sample. The score was compared with other published prediction rules for patients with stable COPD.

**Results:**

In total, 2,487 patients were included in the study. Predictors of death during hospital admission, or within 1 week of discharge to home from the ED were patient age, baseline dyspnea, previous need for long-term home oxygen therapy or non-invasive mechanical ventilation, altered mental status, and use of inspiratory accessory muscles or paradoxical breathing upon ED arrival (area under the curve (AUC) = 0.85). Addition of arterial blood gas parameters (oxygen and carbon dioxide partial pressures (PO_2_ and PCO_2_)) and pH) did not improve the model. The same variables were predictors of death at 1 month (AUC = 0.85). Compared with other commonly used tools for predicting the severity of COPD in stable patients, our rule was significantly better.

**Conclusions:**

Five clinical predictors easily available in the ED, and also in the primary care setting, can be used to create a simple and easily obtained score that allows clinicians to stratify patients with eCOPD upon ED arrival and guide the medical decision-making process.

## Background

Chronic obstructive pulmonary disease (COPD) is a leading chronic condition in many countries [1]. Patients can experience exacerbation of COPD (eCOPD) that often requires assessment in an emergency department (ED) and hospitalization. Exacerbations play a major role in the burden of COPD, its evolution, and its cost [2,3]. Some exacerbations can be severe, leading to death or the need for intubation, whereas others are more moderate, requiring little more than an adjustment of the patient’s current medical therapy. Currently, ED physicians must rely largely on their experience and the patient's personal criteria for gauging how an eCOPD will evolve. A clinical prediction rule that could help predict eCOPD evolution would allow ED physicians to make better informed decisions about treatment.

A couple of severity scores have been developed for patients with stable COPD [4,5], but these do not apply to patients with eCOPD. Several studies have attempted to develop severity scores for eCOPD, but none has been adopted by clinicians, largely because they do not include important clinical variables or they have other methodological problems [6-10]. Several key issues affect the development of severity scores for patients with eCOPD being evaluated in an ED. To date, most such studies have included only patients who are admitted to the hospital, which excludes a significant percentage of patients with eCOPD [11,12]. Another limitation is the choice of reliable predictors that can easily be gathered in the ED. For a clinical prediction rule to be adopted, its development must follow strict methodological norms and must be based on easily available parameters [13,14].

The goal of this study was to develop a clinical prediction rule for short-term death following eCOPD. We defined 'short term 'as any time during the hospital admission or within 1 week after discharge from the ED to home. Data for developing this rule were collected from a large prospective cohort of patients with eCOPD attending a number of different EDs.

## Methods

This prospective cohort study covered 16 hospitals belonging to the Spanish National Health Service (Hospital Costa del Sol, Hospital Valme, Hospital de Motril, Corporació Sanitaria Parc Taulí, Hospital del Mar, Hospital Universitario de La Princesa, Hospital Universitario Gregorio Marañón, Hospital Universitario La Paz, Hospital de Móstoles, Hospital Marqués de Valdecilla, Hospital Santa Marina, Hospital San Eloy, Hospital Galdakao-Usansolo, Hospital Txagorritxu, Complejo Hospitalario Donostia, and Hospital Cruces). The Institutional Review Boards of the participating hospitals approved this project. Patients with eCOPD attending the EDs of any of these hospitals were informed of the goals of the study, and invited to voluntarily participate and sign an informed consent form. All information was kept confidential. Recruitment started in June 2008 and ended in September 2010. A description of the study protocol was published previously [15].

Patients were eligible for the study if they presented to the ED with symptoms consistent with eCOPD. COPD was confirmed if the patient had a forced expiratory volume in 1 second/forced vital capacity (FEV_1_/FVC) quotient of less than 70%. Exacerbation was defined as an event in the natural course of the disease characterized by a change in the patient’s baseline dyspnea, cough, and/or sputum that was beyond normal day to day variations and may have warranted a change in regular medication in a patient with underlying COPD [16]. For cases of COPD newly diagnosed in the ED to be included in the study, they had to be confirmed by spirometry within 60 days after the index episode at a time when the patient was stable [17]. Patients were excluded from the study if, at the time they were seeing at the ED, they had eCOPD complicated by a comorbidity such as pneumonia, pneumothorax, pulmonary embolism, lung cancer, or left cardiac insufficiency. Other exclusion criteria included a diagnosis of asthma, extensive bronchiectasis, sequelae of tuberculosis, pleural thickening, or restrictive disease. Patients who did not wish to participate were also excluded.

### Data collected

Data collected upon arrival in the ED included socioeconomic data, information about the patient’s respiratory function (arterial blood gases, respiratory rate, dyspnea), consciousness level measured by the Glasgow Coma Scale (GCS; altered consciousness defined as a score of ≤15 points, unaltered consciousness as a score of >15) [18], and presence of other pathologies recorded in the Charlson Comorbidity Index [19]. Additional data collected in the ED at the time a decision was made to admit or discharge the patient included the patient’s symptoms, signs, and respiratory status at that time. All information regarding the ED evaluation was recorded as it was provided by the ED physician in charge of the patient.

For patients admitted to the hospital, we collected additional data from the patient’s medical record and from a direct interview with the patient on the first day after admission and on the day of discharge. For patients discharged from the ED to home, telephone interviews were conducted with the patient around 1 and 7 days after discharge to assess hospital readmission and vital status. We asked all patients to tell us about their physical activity, general health, and dyspnea level while in stable condition before the eCOPD index and at 24 hours after being admitted to the hospital or discharged from the ED to home. We used the Medical Research Council (MRC) breathlessness scale [20] to measure baseline dyspnea.

For all patients with known COPD, additional variables were collected from medical records, including baseline severity of COPD as measured by FEV_1_; hospital admissions for eCOPD during the previous 12 months; baseline therapy (inhaled short-acting or long-acting beta-agonist, short-acting or long-acting anticholinergics, oral or inhaled corticosteroid, theophyllines, and/or need for noninvasive mechanical ventilation (NIMV) or long-term home oxygen therapy (LTHOT)); and presence of diabetes, hypertension, ischemic heart disease and/or valve disease, cor pulmonale, hepatic disease, peptic ulcer disease, psychiatric disorders, rheumatic disease, and any history of stroke or deep-vein thrombosis, and of other conditions needed to determine the Charlson Comorbidity Index.

Reviewers were trained before data collection, and a precise manual was developed, which was closely followed for the collection of data.

### Definitions of outcome measure

The main outcomes measured were death occurring during the hospital admission or within 1 week of discharge to home from the ED, and death within 1 month of the index ED visit. Additional outcomes reported in this study were admission to the hospital and, if admitted, length of hospital stay; admission to the intensive care unit (ICU); need for invasive mechanical ventilation (IMV); need for NIMV for 2 or more days when mechanical ventilation was not used at home before admission; and admission to an intermediate respiratory care unit (IRCU) for 2 or more days (a minimum of 2 days was chosen to include only those patients needing more intensive and prolonged therapeutic interventions).

Patients were followed by phone or direct interview to reduce losses to a minimum.

### Statistical analysis

The unit of the analysis was the patient. For patients who had more than one eCOPD requiring an ED visit during the recruitment period, only the first visit was considered for the analysis. Assumptions about how missing data were handled in this study have been described elsewhere. In general, missing values were imputed. In the case of missing data on basal level of dyspnea (MRC classification) it was imputed because class 5 comparison of the mortality rate of this group of patients with the other MRC categories (1 to 4) gave *P* values of less than 0.0001, whereas the *P* value within the MRC group 5 was 0.63.

The total sample was randomly divided in two to give a derivation sample and a validation sample. Descriptive analyses for both samples included frequency and percentages for categorical variables, and mean and standard deviations for continuous variables. The χ^2^and Fisher’s exact tests were used to test for statistical significance between proportions. For continuous variables, the Wilcoxon U-test was used.

In order to identify risk factors associated with short-term mortality in COPD, we performed univariate analyses in the derivation sample using logistic regression. Variables that were significant at *P* = 0.20 were entered into a multivariate logistic regression model. We performed logistic regression models in the derivation sample to select separately the variables for prediction of death. Final predictive factors in the multivariate analysis were those with a significance level of 0.05. Beta estimates, odds ratios (ORs) and 95% confidence intervals (95% CIs) were provided for the multivariate analysis. We developed a score by assigning a weight to each risk factor category based on the β parameter from the multivariate logistic regression. From the continuous score, four risk categories were created (mild, moderate, severe, and very severe). We considered the optimal classifier point as the point that maximized the sum of sensitivity and specificity. Final models were also adjusted by the treating hospital to see if that affected the results.

The predictive accuracy of each model was determined by calculating the area under the receiver operating characteristic (ROC) curve (discrimination) (AUC), and the models were calibrated by means of the Hosmer and Lemeshow test. We validated all AUCs—that is, those from the model and those from the continuous and categorical scores—in the validation sample by deriving the AUC in this sample [21].

Additional multivariate logistic regressions models were performed to evaluate the impact on short-term mortality of arterial blood gas values (pH, PCO_2_ and PO_2_) measured both at the time the patient arrived in the ED and at the time a decision was made to hospitalize the patient or discharge them to home from the ED. These were adjusted by our categorical score.

We compared various outcomes between the four risk classes of our categorical score. These included ICU admission; need for IMV; admission to an IRCU; admission to the hospital and, if admitted, length of hospital stay; readmissions within 10 days, 1 month, and 2 months after the index ED visit; and subsequent ED visits in the 2 months following the index ED visit.

To compare the predictive ability of our score, we applied information from all patients to previously created predictor scores for mortality in patients with stable COPD. These included the ADO (age, baseline dyspnea, and airflow obstruction measured by FEV1%) index [22], the HADO (health, activity, dyspnea, and airflow obstruction) score [23], baseline FEV1% classified according to the Global Initiative for Chronic Obstructive Lung Disease (GOLD) standards (FEV1% alone) [16], and the GOLD COPD combined assessment (baseline dyspnea plus previous exacerbations over the previous year) [24]. For the latter, we used hospitalizations during the previous year, as we did not collect the variable 'previous exacerbations.' Descriptive statistics for the previous mortality predictor scales (FEV1% classifications) were computed, and each AUC and its CI were determined and compared with our score.

All effects were considered significant at P < 0.05, unless otherwise stated. All statistical analyses were performed using SAS for Windows statistical software, v9.2 (SAS Institute, Inc., Carey, NC) and R^©^ software v2.13.0.

## Results

A total of 3,276 episodes were assesses for the study. Of these, 198 (6%) were excluded because COPD was complicated by other major pathologies at the time of ED admission (cardiovascular conditions: 59 (29.8%); pneumonia: 55 (27.8%); cancer: 21 (10.6%); other respiratory problems, 13 (6.6%); and other conditions, 50 (25.2%)). A further 56 episodes were excluded because the COPD diagnosis was not confirmed by spirometry within 60 days of the index episode. Another 145 episodes were lost because of incomplete data without the possibility of retrieving the information needed for the study. Finally, 390 episodes were excluded because the patient had more than one ED visit. No differences were observed in mortality (4.4% *versus*. 2.7%; *P* = 0.32) or other parameters, such as arterial blood gas as pH upon ED arrival (*P* = 0.52) between episodes excluded because patients had multiple eCOPD episodes and episodes included from patients who had only a single eCOPD episode.

The final population included 2,487 patients. Of these, 1,537 (61.8%) were admitted to the hospital and 950 (38.2%) were discharged from the ED to home.

Comparison of the derivation and validation samples is shown in Table 1. Variables related to short-term mortality in the univariate analysis of the derivation sample are displayed in Table 2. Variables that were also evaluated, but were not statistically significantly correlated with outcome, included previous oral corticosteroid use, chronic renal disease, cerebrovascular disease, chronic liver disease, edema at ED admission, and four variables recorded upon ED arrival:O_2_ saturation, blood pressure, temperature, and serum glucose.

**Table 1:** Descriptive characteristics of derivation and validation samples

	**Derivation**		**Validation**		***P *** **value**
	**N**	**n (%)**^**a**^	**N**	**n (%)**^**a**^	
Age, years^b^	1242	72.32 ± 9.81	1244	73.23 ± 9.51	0.02
Male sex	1242	1136 (91.47)	1242	1131 (91.06)	0.72
Baseline FEV	1046		1023		
≥50		367 (35.09)		352 (34.41)	0.75
<50		679 (64.91)		671 (65.59)	
Charlson Comorbidity Index^b^	1243	2.25 ± 1.55	1244	2.89 ± 1.6	0.79
Previous use of LTHOT or NIMV at home	1243	424 (34.11)	1244	417 (33.52)	0.76
Number of COPD-related admissions in the previous 12 months^bd^	1228	0.84 ± 1.37	1223	0.83 ± 1.38	0.65
Altered consciousness	1242	31 (2.50)	1243	39 (3.14)	0.33
Presence of edema	1164	225 (19.33)	1168	211 (18.07)	0.43
Dyspnea upon ED arrival	1182	803 (67.94)	1179	798 (67.68)	0.9
Heart rate upon ED arrival^b^	1168	95.51 ± 19.35	1163	94.32 ± 18.69	0.1
Use of inspiratory accessory muscle upon ED arrival	1243	248 (19.95)	1244	262 (21.06)	0.49
Breathing frequency upon ED arrival	1135		1132		0.8
<20 breaths/min		186 (16.39)		188 (16.61)	
20 to 24 breaths/min		506 (44.58)		489 (43.2)	
>24 breaths/min		443 (39.03)		455 (40.19)	
pH upon ED arrival	1147		1148		0.3
≥7.35		986 (85.96)		1005 (87.54)	
7.26 to 7.35		136 (11.86)		114 (9.93)	
<7.26		25 (2.18)		29 (2.53)	
PO_2_ upon ED arrival	1137		1141		0.77
>60 (O_2_ saturation >90%)		563 (49.52)		571 (50.04)	
45 to 60 (O_2_ saturation <90%)		420 (36.94)		427 (37.42)	
≤45		154 (13.54)		143 (12.53)	
PCO_2_ upon ED arrival	1078		1076		0.65
≤45		603 (55.94)		629 (58.46)	
45 to 55		249 (23.1)		235 (21.84)	
55 to 65		127 (11.78)		114( 10.59)	
>65		99 (9.18)		98 (9.11)	
Baseline dyspnea (MRC scale)	1243		1244		0.13
Missing		121(9.73)		129(10.37)	
Grade 1		87 (7)		101 (8.12)	
Grade 2		321 (25.82)		279 (22.43)	
Grade 3		247 (19.87)		254 (20.42)	
Grade 4		345 (27.76)		327 (26.29)	
Grade 5		122 (9.81)		154 (12.38)	
Outcomes					
Short-term mortality^c^	1243	30 (2.41)	1244	29 (2.33)	0.89
1-month mortality	1243	40 (3.22)	1244	48 (3.86)	0.39

*Abbreviations*: *COPD* chronic obstructive pulmonary disease, *ED* emergency department, *FEV* forced expiratory volume, *LTHOT* long-term home oxygen therapy, *NIMV* non-invasive mechanical ventilation, *PCO*_*2*_ carbon dioxide partial pressure, *PO*_*2*_ oxygen partial pressure.

^a^Unless otherwise specified.

^b^Mean ± SD.

^c^During hospital admission or within 1 week of discharge to home from the ED.

^d^Number of COPD-related admissions in the previous 12 months, median (25th to 75th percentile): 0 (0–1) in both samples.

**Table 2:** Predictors of short-term mortality and 1 month mortality in the derivation sample

**Parameter**	**Short-term mortality**^**a**^		**1 month mortality**	
	**OR**	***P *** **value**	**OR**	***P *** **value**
Age, years^b^	1.051	0.0237	1.0411	0.0294
Male sex	1.314	0.7120	1.8	0.4226
Baseline FEV: <50 *versus* ≥50	2.197	0.1185	2.587	0.0365
Charlson Comorbidity Index^b^	1.297	0.0016	1.360	<0.0001
Previous use of LTHOT or NIMV at home	4.690	0.0001	4.755	<0.0001
Number of COPD-related admissions in the previous 12 months^c^	1.168	0.1502	1.137	0.187
Altered consciousness	11.87	<0.0001	8.308	<0.0001
Presence of edema	1.056	0.9140	1.246	0.6268
Dyspnea upon ED arrival	3.531	0.0414	1.92	0.1268
Heart rate upon ED arrival^b^	1.028	0.0017	1.020	0.0128
Use of inspiratory accessory muscle upon ED arrival	4.026	0.0001	2.79	0.0019
Breathing frequency upon ED arrival				
20 to 24 *versus* <20	3.350	0.2530	2.811	0.1726
>24 *versus* <20	7.381	0.0529	4.123	0.0585
pH upon ED arrival				
7.26 to 7.35 *versus* ≥7.35	2.631	0.0456	2.086	0.0931
<7.26 *versus* ≥7.35	7.777	0.0020	7.323	0.0006
PO_2_ upon ED arrival				
45 to 60 *versus* >60	0.762	0.5431	0.630	0.2350
≤45 *versus* >60	1.046	0.9379	0.866	0.7764
PCO_2_ upon ED arrival				
46 to 55 *versus* ≤45	2.8226	0.047	3.003	0.0115
56 to 65 *versus* ≤45	0.676	0.7150	0.949	0.9463
>65 *versus* ≤45	8.514	<0.0001	7.413	<0.0001
Baseline dyspnea (MRC scale)				
MRC3 *versus* MRC1 to MRC2	6.398	0.1064	3.335	0.2960
MRC4 *versus* MRC1 to MRC2	8.035	0.0397	4.810	0.0601
MRC5 *versus* MRC1 to MRC2	61.599	<0.0001	26.115	<0.0001
Missing *versus* MRC1 to MRC2	50.583	<0.0001	22.185	<0.0001

*Abbreviations*: *COPD* chronic obstructive pulmonary disease, *ED* emergency department, *FEV* forced expiratory volume, *LTHOT* long-term home oxygen therapy, *MRC* Medical Research Council, *NIMV* non-invasive mechanical ventilation, *PCO*_*2*_ carbon dioxide partial pressure, *PO*_*2*_ oxygen partial pressure.

^a^Short-term mortality: mortality during hospital admission or, if discharged from the ED, in 1 week.

^b^ Variables included as continuous.

All variables with *P* values below 0.20 were entered into a multivariate logistic regression model. Five parameters shaped the final model for predicting death during hospital admission or within 1 week of discharge from the ED to home: age; previous history of LTHOT or NIMV; altered consciousness measured by GCS; use of accessory inspiratory muscles or paradoxical breathing upon ED arrival; and baseline dyspnea measured by the MRC scale (model AUC = 0.85, 95% CI 0.77 to 0.93). In separate models, we evaluated the effect on the final model of the role of arterial blood gases by adding the values of PO_2_, PCO_2_, and pH measured at both ED arrival and at the time that a decision was made to hospitalize the patient or discharge them from the ED to home. None of these was statistically significant.

**Figure 1:**
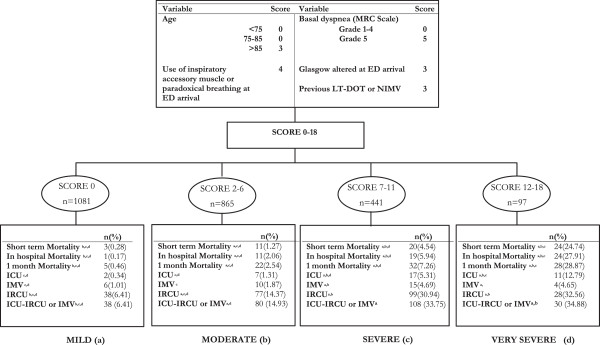
**Death in exacerbation of chronic obstructive pulmonary disease: construction of continuous and categorical scores and relation with different outcomes. **ICU: Intensive Care Unit, IMV: Invasive mechanical ventilation, IRCU: Intermediate Respiratory Care Unit, LT-HOT: Long-term home oxygen therapy, NIMV: Noninvasive mechanical ventilation. Superscript letters indicated statistical significant differences among the DeCOPD risk classes for the outcomes displayed.

We assigned a score to each category of the variables selected in the model based on their parameter estimates. Adding these individual scores yielded a final risk score that we called the continuous death in eCOPD (DeCOPD) risk score (AUC = 0.85, 95% CI 0.77 to 0.93) (Table 3). A categorical DeCOPD score was created by dividing the continuous DeCOPD score into four risk categories (mild, moderate, severe, and very severe) based on the risk of experiencing the outcome (Figure 1). This yielded an AUC of 0.84 (95% CI 0.76 to 0.91). Hosmer-Lemeshow tests were all *P* > 0.62. The continuous DeCOPD score (AUC = 0.84; 95% CI 0.77 to 0.92) and the categorical DeCOPD risk classes (AUC = 0.85; 95% CI 0.78 to 0.92) were validated in a separate sample (validation sample). Introducing the treating hospital variable into any model did not change the model, nor was the individual hospital significantly related to mortality (*P* = 0.064 unadjusted; *P* = 0.96 adjusted by the DeCOPD continuous score).

**Table 3:** Predictors of short-term mortality and 1 month mortality in the derivation sample

**Outcome prediction model/parameter**	**Estimate**	**OR**	**95% CI of the AUC**	***P *** **value**	**Score**
Short-term mortality^a^					
Age, years					
75 to 85 *versus* <75	0.2417	1.273	0.540 to 3.003	0.5808	0
>85 *versus* <75	1.3062	3.692	1.134 to 12.022	0.0301	3
Previous use of LTHOT or NIMV at home	1.1557	3.176	1.278 to 7.893	0.0128	3
Altered consciousness	1.1852	3.271	1.031 to 10.376	0.0442	3
Use of inspiratory accessory muscle or paradoxical breathing upon ED arrival	1.4740	4.367	1.912 to 9.972	0.0005	4
Baseline dyspnea (MRC scale)					
Grade 5 *versus* grades 1 to 4	2.0456	7.734	2.923 to 20.463	<0.0001	5
Missing *versus* grades 1 to 4	2.5581	12.912	4.654 to 35.821	<0.0001	5
^b^Derivation sample: AUC_model_ = 0.85 (0.77 to 0.93); AUC_score_ = 0.85 (0.77 to 0.93) AUC_score _ cat_ = 0.84 (0.76 to 0.91)					
^b^Validation sample: AUC_model_ = 0.88 (0.82 to 0.94); AUC_score_ = 0.84 (0.77 to 0.92) AUC_score _ cat_ = 0.85(0.78 to 0.92)					
1 month mortality (validation sample)					
Age, years					
75 to 85 *versus* <75	0.8199	2.270	1.092 to 4.719	0.0281	
>85 *versus* <75	1.9253	6.857	2.701 to 17.411	<0.0001	
Previous use of LTHOT or NIMV at home	1.3737	3.950	1.956 to 7.976	0.0001	
Altered consciousness	0.9513	2.589	0.887 to 7.557	0.0817	
Use of inspiratory accessory muscle or paradoxical breathing upon ED arrival	1.2508	3.493	1.850 to 6.596	0.0001	
Baseline dyspnea (MRC scale)					
Grade 5 *versus* grades 1 to 4	1.1743	3.236	1.581 to 6.625	0.0013	
Missing *versus* grades 1 to 4	1.3324	3.790	1.643 to 8.742	0.0018	
^b^Validation sample: AUC_model_ = 0.85 (0.81 to 0.90); AUC_score_ = 0.83 (0.78 to 0.88); AUC_score _ cat_ = 0.83 (0.78 to 0.88)					

*Abbreviations*: *AUC* area under the receiver operating characteristic curve, *LTHOT* long-term home oxygen therapy, *MRC* Medical Research Council, *NIMV* non-invasive mechanical ventilation.

^a^Short-term mortality: mortality during hospital admission or, if discharged from the ED, in 1 week.

^b^AUCs are the results for the whole model, the continuous score, and the categorical score, respectively.

The 1-month mortality was tested in the same model. It had an AUC of 0.85 (95% CI 0.81 to 0.90); AUCs were 0.83 (95% CI 0.78 to 0.88) for the continuous DeCOPD score and 0.83 (95% CI 0.78 to 0.88) for the categorical DeCOPD score.

In addition to short-term mortality, other outcomes that reflect adverse eCOPD evolution (for example, need for IMV, admission to ICU or IRCU) were compared between the four risk classes. Differences were observed across most of the categories for such outcomes (Figure 1). Specifically, differences between the four risk classes were observed for short-term mortality, in-hospital mortality, and 1 month mortality. When the four classes were dichotomized in two (score ≥12) the accuracy results for short-term mortality were: sensitivity 41%, specificity 97%, positive predictive value (PPV) 25%, and negative predictive value (NPV) 99%. When the cut-off point was 7 or above, the accuracy results for ICU admission, IMV use, or IRCU admission were: sensitivity 53.9%, specificity 79.05%, PPV 34%, and NPV 89.54%. The relationship of the DeCOPD categorical score in two categories (score ≥7) with other outcomes (for example, hospital admission, length of stay, readmissions or new ED visits) is presented (see Additional file 1).

Finally, we compared the continuous and categorical DeCOPD scores with scores created for patients with stable COPD, including the ADO index, HADO score, GOLD FEV1% and GOLD COPD combined assessment. The AUC of our score was always superior to and statistically significantly different from these other assessments (Table 4).

**Table 4:** Comparison of different prediction scales on short-term mortality on patients with eCOPD

**Outcome**	**N**	**Short-term mortality**		***P *** value*	**AUC**	**95% CI**	***P *** value**
		**Yes**	**No**				
DeCOPD score mean (sd)	2484	9.03 (4.27)	3.07 (3.4)	≤0.0001	0.85	0.79 to 0.90	Ref^a^
DeCOPD score (categorical)	2484			≤0.0001	0.84	0.79 to 0.89	0.38
Risk							
Mild	1081	3 (0.28)	1078 (99.72)				
Moderate	865	11 (1.27)	854 (98.73)				
Severe	441	20 (4.54)	421 (95.46)				
Very severe	97	24 (24.74)	73 (75.26)				
GOLD FEV1%	2069			0.0133	0.62	0.56 to 0.69	<0.0001
FEV1% ≥80	76	0 (0.0)	76 (100)				
50 ≤ FEV1% <80	643	6 (0.93)	637 (99.07)				
30 ≤ FEV1% <50	959	28 (2.92)	931 (97.08)				
FEV1% ≤30	391	13 (3.32)	378 (96.68)				
GOLD COPD combined assessment	2090			<0.002	0.71	0.67 to 0.75	<0.0001
Low risk, low symptom burden	286	1 (0.35)	285 (99.65)				
Low risk, higher symptom burden	335	4 (1.19)	331 (98.81)				
High risk, low symptom burden	351	0 (0.00)	351 (100)				
High risk, higher symptom burden	1118	46 (4.11)	1072 (95.89)				
HADO score	1887			0.0002	0.68	0.63 to 0.72	<0.0001
Mild (<4)	188	0	188 (100)				
Moderate (5 to 7)	604	3 (0.50)	601 (99.50)				
Severe (>8)	1095	33 (3.01)	1062 (96.99)				
ADO index (0 to 14) mean (sd)	2067	11.36 (1.71)	9.29 (2.19)	≤0.0001	0.78	0.71 to 0.84	0.05

Abbreviations: ADO age, baseline dyspnea, and airflow obstruction; AUC, area under the curve; COPD, chronic obstructive pulmonary disease; DeCOPD, Death in exacerbation of chronic obstructive pulmonary disease; ED, emergency department; FEV, forced expiratory volume; GOLD, Global Initiative for Chronic Obstructive Lung Disease; HADO, health, activity, dyspnea, and airflow obstruction; LTHOT; long-term home oxygen therapy; MRC, Medical Research Council; NIMV, non-invasive mechanical ventilation; PCO_2_ carbon dioxide partial pressure; PO_2_, oxygen partial pressure.

^a^Reference value.

*p-value of the relationship of each parameter with short-term mortality.

**p-value or the comparison of areas under Receiver Operating Characteristic Curves with respect to the AUC of the DECOPD continuous scale.

## Discussion

This study describes the development of a clinical prediction rule for short-term mortality in patients with exacerbations of COPD, derived from a large, multicenter prospective cohort. This rule predicts death using clinical data generally available in the ED, and also often available in the primary care setting. Development of this rule followed proper procedures for derivation and validation [11,12], and the rule provides excellent predictive validity.

Given how common exacerbations are among patients with COPD, and the substantial effects of these exacerbations on health, health-related quality of life, and healthcare costs, predicting the severity of exacerbations could significantly improve both care and allow more targeted allocation of healthcare resources. To date, however, no validated clinical prediction rules are available to stratify all patients experiencing an eCOPD upon their arrival in an ED, or to provide valid and reliable clinical rules or scores for predicting short-term outcomes [6,9].

To the best of our knowledge, no prior prospective cohort studies including both patients admitted to the hospital and those discharged to home from the ED have been employed to develop and validate a clinical prediction rule for death using variables commonly available in the ED [6,9]. Although previous authors have proposed predictive models for death or ICU admission, and some have created severity scores for patients with eCOPD, these did not evaluate such a complete range of variables as in our study, including arterial blood gases and other relevant data from the ED, and this limits their results [7-9,25,26].

The five factors in our final model—age, previous history of LTHOT or NIMV, use of inspiratory accessory muscles or paradoxical breathing upon ED arrival, altered mental status, and baseline dyspnea—have been observed separately as predictors of poor outcomes in previous studies [6,8,9,11,26-28]. Age is always a surrogate of other still unknown variables. Previous use of LTHOT or NIMV and the baseline level of dyspnea reflect the basal severity of the patient’s COPD. Altered mental status or the use of inspiratory accessory muscles upon arrival in the ED indicate acute cardiopulmonary compromise of the current presentation. Some of the parameters included in our rule are not modifiable, such as age or previous need of LTHOT or NIMV at home, which reflect the fragility of the patient and their disease. Severe baseline dyspnea can be modified through respiratory rehabilitation programs. Even for patients with severe COPD who cannot undertake respiratory rehabilitation, efforts to avoid new exacerbations or readmissions can be conducted through programs such as telemedicine or continuity of care, as has been reported elsewhere [29]. Finally, use of inspiratory accessory muscles or paradoxical breathing and altered level of consciousness reflect the severity of the current exacerbation. These are not permanent but modifiable and, in conjunction with other factors, should alert the ED physician to the severity of the patient’s eCOPD and guide appropriate treatment and adequate follow-up.

We found that measurement of arterial blood gases upon ED arrival or at the time a decision is made to hospitalize the patient or discharge them to home from the ED did not add any value to our score. We therefore did not include this measurement in our final model due to the high rate of missing data for this parameter, mainly at ED decision time. Nevertheless, future studies should evaluate the final role of these parameters in our score, although room for improvement in its predictive ability appears to be low.

From a practical point of view, our DeCOPD risk classification allows a clinician to identify patients who could be discharged to home (those with mild eCOPD) from those who should be admitted (severe and very severe eCOPD) because the latter are more likely to have an adverse event or to need intensive treatment and closer follow-up. For patients in the moderate category, further clinical information would be needed in the ED to decide if they should be admitted and if they need intensive treatment.

We compared our score with other rules or parameters used to classify patients with COPD, such as the ADO index, HADO score, GOLD FEV1%, and GOLD COPD combined assessment [16,21,22,24]. In each case, these other scores performed poorly compared with our score. However, we must point out that those rules were created for patients with stable COPD, not those experiencing exacerbations. Tabak *et al*. recently proposed a prediction model derived from a large administrative database of hospitalized patients; their model has AUCs around 0.83, also but requires vital signs upon admission and laboratory results [10].

Our study has several strengths. The prediction rule we propose was created from data prospectively collected from a large (n = 2,487) group of patients experiencing COPD exacerbations recruited from 16 different EDs, each with its own set of guidelines or clinical practices for evaluating and treating patients with eCOPD. This is likely to improve the generalizability of the results. The cohort included both patients admitted to the hospital and patients who were discharged to home. Furthermore, we collected a broad range of clinical variables, many of them from the ED, and tested them in different models. We also strove to properly validate these prediction rules, following best practices for such studies [13,14].

The lack of data for some key variables is the main limitation of our work. In a study such as this, clinical practice in the ED prevails over research requirements. We did not ask ED physicians to modify their clinical practices in any way. Thus, we had to work with the information available. Occasionally this led to missing data, but this did not appear to affect the results. In the case of baseline dyspnea (measured by the MRC scale), for example, we determined that patients with missing data had similar outcomes to those with class 5 baseline dyspnea, and the AUC of the final model was not affected by leaving these patient in the calculations or removing them.

It must also be noted that the patient population was almost entirely comprised of men (97%). Similar gender distributions have been observed in other studies performed in our country [30], which probably reflects the smoking patterns in Spain in the mid-20th century. Although we do not consider this to be a serious limitation, it could affect the generalizability of the results.

Finally, we did not include any biomarkers. At the time this study was conducted, several biomarkers were being evaluated for eCOPD (C-reactive protein, procalcitonin, copeptin, pro-adrenomedullin, pro-endothelin, and B-type natriuretic peptide). However, these were not routinely employed in the ED or in the hospital [31]. Whether these biomarkers add value to scores based on clinical variables will be the aim of future studies.

## Conclusions

We developed a clinical prediction rule for a critical outcome, death, for patients attending an ED with eCOPD. The five variables included in our model are easily available in the ED (and also in primary care settings) and the score is easy to estimate. This clinical prediction rule could be employed in the clinical management of patients with eCOPD to guide their treatment and follow-up by in the ED and also by primary care physicians. Future studies are needed for validation of this prediction rule and to further demonstrate its value in clinical practice.

## Competing interests

No conflicts of interest exist in this study.

“All authors have completed the Unified Competing Interest form at http://www.icmje.org/coi_disclosure.pdf (available on request from the corresponding author) and declare that (1) none of the authors have support from any company for the submitted work; (2) none of the authors] have any relationships with any company that might have an interest in the submitted work in the previous 3 years; (3) their spouses, partners, or children do not have any financial relationships that may be relevant to the submitted work; and (4) none of the authors have any non-financial interests that may be relevant to the submitted work.”

## Authors’ contributions

JMQ: Conception, hypothesis delineation, and design of the study; acquisition of the data and interpretation of the results; and writing the article and its revision prior to submission. CE: Conception, hypothesis delineation, and design of the study; acquisition of the data and interpretation of the results; and writing the article and its revision prior to submission. AU: Conception, hypothesis delineation, and design of the study; analysis and interpretation of the results; and writing the article and its revision prior to submission. IB: Conception, hypothesis delineation, and design of the study; analysis and interpretation of the results; and writing the article and its revision prior to submission. SG: Conception, hypothesis delineation, and design of the study; acquisition of the data and interpretation of the results; and revision of the article prior to submission. NG: Conception, hypothesis delineation, and design of the study; acquisition of the data and interpretation of the results; and revision of the article prior to submission. IA: Conception, hypothesis delineation, and design of the study; analysis and interpretation of the results; and writing the article and its revision prior to submission. IL: Conception and design of the study; acquisition of the data; and revision of the article prior to submission. MB: Conception and design of the study; acquisition of the data; and revision of the article prior to submission. NF: Conception and design of the study; acquisition of the data; and revision of the article prior to submission. SV: Conception and design of the study; acquisition of the data; and revision of the article prior to submission. All authors read and approved the final manuscript.

JMQ had full access to all of the data in the study and takes responsibility for the integrity of the data and the accuracy of the data analysis.

The IRYSS- COPD group included the following co-investigators: Dr Jesús Martínez-Tapias (Dirección Económica, Área Gestión Sanitaria Sur Granada); Alba Ruiz (Hospital de Motril, Granada); Dr Eduardo Briones, Dr Silvia Vidal (Unidad de Calidad, Hospital Valme, Sevilla); Dr Emilio Perea-Milla, Francisco Rivas (Servicio de Epidemiología, Hospital Costa del Sol, Málaga - CIBER Epidemiología y Salud Pública-CIBERESP); Dr Maximino Redondo (Servicio de Laboratorio, Hospital Costa del Sol, Málaga-CIBERESP); Javier Rodríguez Ruiz (Responsable de Enfermería del Área de Urgencias, Hospital Costa del Sol, Málaga); Dr Marisa Baré (Epidemiología y Evaluación, Corporació Sanitaria Parc Taulí-CSPT, Sabadell), Dr Manel Lujan, Dr Concepción Montón, Dr Amalia Moreno, Dr Josune Ormaza, Dr Javier Pomares (Servicio de Neumología, CSPT); Dr Juli Font (Medicina, Servicio de Urgencias; CSPT), Dr Cristina Estirado, Dr Joaquín Gea (Servicio de Neumología, Hospital del Mar, Barcelona); Dr Elena Andradas, Dr Juan Antonio Blasco, Dr Nerea Fernández de Larrea (Unidad de Evaluación de Tecnologías Sanitarias, Agencia Laín Entralgo, Madrid); Dr Esther Pulido (Servicio de Urgencias, Hospital Galdakao-Usansolo, Bizkaia); Dr Jose Luis Lobo (Servicio de Neumología, Hospital Txagorritxu, Araba); Dr Mikel Sánchez (Servicio de Urgencias, Hospital Alto Deba, Gipuzkoa); Dr Luis Alberto Ruiz (Servicio de Respiratorio, Hospital San Eloy, Bizkaia); Dr Ane Miren Gastaminza (Hospital San Eloy, Bizkaia); Dr Ramon Agüero (Servicio de Neumología, Hospital Marques de Valdecilla, Santander); Dr Gabriel Gutiérrez (Servicio de Urgencias, Hospital Cruces, Bizkaia); Dr Belén Elizalde (Dirección Territorial de Gipuzkoa); Dr Felipe Aizpuru (Unidad de Investigación, Hospital Txagorritxu, Álava/CIBER Epidemiología y Salud Pública-CIBERESP); DrInmaculada Arostegui (Departamento de Matemática Aplicada, Estadística e Investigación Operativa, UPV- CIBER Epidemiología y Salud Pública (CIBERESP); Amaia Bilbao (Fundación Vasca de Innovación e Investigación Sanitarias (BIOEF)/CIBER Epidemiología y Salud Pública-CIBERESP); Dr Eva Tabernero and Carmen M Haro (Hospital de Santa Marina); Dr Cristóbal Esteban (Servicio de Neumología, Hospital Galdakao-Usansolo, Bizkaia); Dr Nerea González, Susana Garcia, Iratxe Lafuente, Urko Aguirre, Irantzu Barrio; Miren Orive, Edurne Arteta, Dr Jose M Quintana (Unidad de Investigación, Hospital Galdakao-Usansolo, Bizkaia/CIBER Epidemiología y Salud Pública-CIBERESP).

## Pre-publication history

The pre-publication history for this paper can be accessed here:

http://www.biomedcentral.com/1741-7015/12/66/prepub

## Supplementary Material

Additional file 1: Table S5 Relationship of the death in exacerbatino of chronic obstructive pulmonary disease (DeCOPD) categorical severity score with other outcomes. Click here for file
