# Supplementary material for: Predictive score for mortality in patients with COPD exacerbations attending hospital emergency departments
Source: BMC Med. 2014 Apr 23;12:66. doi: 10.1186/1741-7015-12-66 (PMC4021970; doi:10.1186/1741-7015-12-66)
Supplement: Additional file 1: Table S5 — Relationship of the death in exacerbatino of chronic obstructive pulmonary disease (DeCOPD) categorical severity score with other outcomes. [file 1741-7015-12-66-S1.docx]

**Table 5**. Relationship of the DeCOPD categorical severity score with other outcomes

|  |  | **DeCOPD categories** | |  | **Accuracy measures** | |
| --- | --- | --- | --- | --- | --- | --- |
| **Variable** | **n** | **Mild- Moderate** | **Severe- Very Severe** | **p-value** | **Sensitivity/**  **Specificity** | **PPV/**  **NPV** |
| Readmission in 2 months | 2484 | 459(23.59) | 172(31.97) | <0.0001 | 0.27/0.8 | 0.32/0.76 |
| Readmission in 1 month | 2484 | 166(8.53) | 56(10.41) | 0.1764 | 0.25/0.78 | 0.1/0.92 |
| Readmission in 10 days | 2484 | 61(3.13) | 22(4.09) | 0.2755 | 0.26/0.79 | 0.4/0.97 |
| New ED visit in 2 months | 2032 | 500(30.10) | 149(40.16) | <0.0001 | 0.26/0.86 | 0.75/0.41 |
| Admission rate to hospital | 2484 | 1129(58.02) | 406(75.46) | 0.0002 | 0.22/0.84 | 0.4/0.7 |
| Length of hospital stay | 1498 | 7.21(5.02) | 9.23(8.57) | <0.0001 |  |  |

PPV: Positive Predictive Value; NPV: Negative Predictive Value
